# Identification of a novel germline *BRCA2* variant in a Chinese breast cancer family

**DOI:** 10.1111/jcmm.14861

**Published:** 2019-11-28

**Authors:** Jingliang Cheng, Jiangzhou Peng, Jiewen Fu, Md. Asaduzzaman Khan, Pingping Tan, Chunli Wei, Xiyun Deng, Hanchun Chen, Junjiang Fu

**Affiliations:** ^1^ Key Laboratory of Epigenetics and Oncology The Research Center for Preclinical Medicine Southwest Medical University Luzhou China; ^2^ Department of Thoracic Surgery The Third Affiliated Hospital of Southern Medical University Guangzhou China; ^3^ Department of Pathology Hunan Cancer Hospital Changsha China; ^4^ Departments of Pathology and Pathophysiology Hunan Normal University School of Medicine Changsha China; ^5^ Department of Biochemistry School of Life Sciences & the State Key Laboratory of Medical Genetics Central South University Changsha China

**Keywords:** BRCA2, breast cancer, genetic counselling, germline, missense mutation, risk factors

## Abstract

Breast cancer is the most frequently diagnosed cancer and the leading cause of cancer‐related deaths in women worldwide. In this study, a large Chinese pedigree with breast cancer including a proband and two female patients was recruited and a familial history of breast cancer was collected by questionnaire. Clinicopathological assessments and neoadjuvant therapy‐related information were obtained for the proband. Blood samples were taken, and gDNA was extracted. The *BRCA1/2* and *PALB2* genes were screened using next‐generation sequencing by a targeted gene panel. We have successfully identified a novel, germline heterozygous, missense mutation of the gene *BRCA2*: c.7007G>T, p.R2336L, which is likely to be pathogenic in the proband and her elder sister who both had breast cancer. Furthermore, the risk factors for developing breast cancer in this family are discussed. Thus, genetic counselling and long‐term follow‐up should be provided for this family of breast cancer patients as well as carriers carrying a germline variant of *BRCA2*: c.7007G>T (p.R2336L).

## 1 INTRODUCTION

Cancer is a major health problem worldwide; in particular, breast cancer is the most frequently diagnosed cancer and the leading cause of cancer‐related death in women based on the GLOBOCAN 2018 estimates in the United States.^1^ In 2019, 1 762 450 new cancer diagnoses and 606 880 cancer deaths are predicted to occur, including approximately 62 930 new cases of female breast cancer in the United States, which accounts for 30% of all new cancer diagnoses in women.^2^ The incidence of breast cancer has been rising for most developing countries in over the last few decades.^3^, ^4^ In China, breast cancer is also the most common cancer in women; more than 1.6 million women have been diagnosed with breast cancer, and 1.2 million women are dying of breast cancer each year.^5^, ^6^ Patients in China currently account for 12.2% of all newly diagnosed breast cancers and 9.6% of all deaths from breast cancer worldwide.^5^

Breast cancer is a multi‐factorial disease that occurs because of various risk factors, such as sex, age, ethnicity, environmental factors, diet and lifestyle.^7^, ^8^ Although non‐hereditary factors are the significant drivers of the observed international and interethnic differences in the disease incidence, genetics, including a personal or family history of breast or ovarian cancer and inherited mutations in *BRCA1* (Breast Cancer 1 gene, OMIM 113705),^9^ *BRCA2* (Breast Cancer 2 gene, OMIM 600185)^10^ and other breast cancer susceptibility genes such as *PALB2* (Partner And Localizer Of BRCA2, OMIM 610355),^11^ account for 6% to 10% of breast cancer cases. A woman's life‐time risk of developing breast and/or ovarian cancer is greatly increased if she inherits a harmful mutation in the genes *BRCA1/2* or *PALB2*. Breast cancer is also influenced by somatic gene mutations and chromosome instability.^12^

In this study, we have successfully identified a germline mutation in the *BRCA2* gene in a large Chinese family with breast cancer, expanding the knowledge and information about genetic mutations related to breast cancer.

## 2 MATERIALS AND METHODS

### 2.1 Ethical statement, patient information and DNA preparation

**Figure 1:**
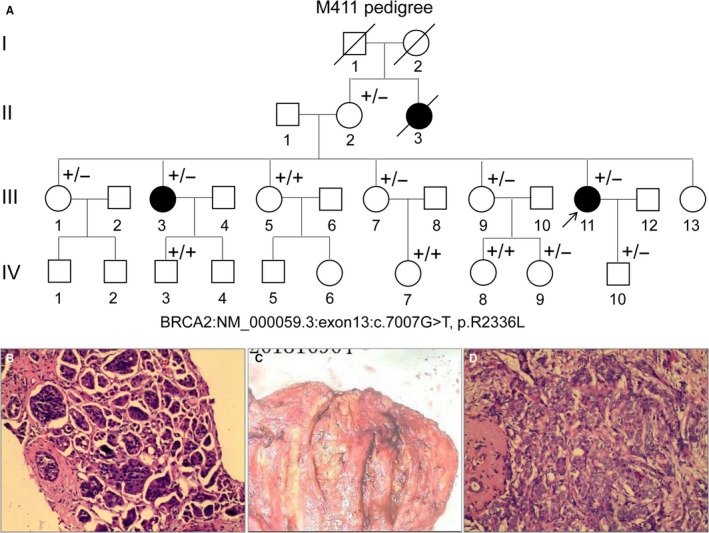
An M411 pedigree with breast cancer. A, M411 pedigree with breast cancer. Normal individuals are shown as clear circles (females) or clear squares (males), whereas affected females are shown as filled circles. The patient with the arrow is the proband (II: 11) with the heterozygous variant of the *BRCA2* gene: NM_000059.3:exon13:c.7007G>T. ‘−’: c.7007G>T variant of *BRCA2*, whereas ‘+’: wild‐type allele of *BRCA2*. B, Representative HE staining results for right breast biopsies with invasive carcinoma from the proband. C, Surgically resected specimen from the proband. D, Representative HE staining results of the surgically resected specimen from the proband

This study was approved by the Ethical Committees in the Southwest Medical University of China with written informed consent from the participants and was conducted in line with the tenets of the Declaration of Helsinki (2013 version). A Chinese pedigree of breast cancer including a proband (Figure 1, pedigree III: 11, arrow) was recruited, and the family history of breast cancer was collected by questionnaire. Clinicopathological assessments and neoadjuvant therapy information were obtained for the proband, such as haematoxylin and eosin (HE) staining, immunohistochemistry (IHC) and fluorescence in situ hybridization (FISH). Fresh peripheral blood samples (2 ml each) were taken from each individual, and gDNA was extracted using our previously described phenol/chloroform method.^13^, ^14^, ^15^ Blood samples from one hundred healthy individuals were taken for DNA extraction as controls.

### 2.2 Targeted NGS, Sanger sequencing and co‐segregation analysis

All of the exons and exon‐intron boundaries of the *BRCA1/2* and *PALB2* genes were screened with next‐generation sequencing (NGS) by a targeted gene panel using an Illumina HiSeq × 10 NGS platform (Illumina, CA, US). Gene capture was up to 500 × depth and had greater than 99% coverage. For mutation identification, the sequenced results were aligned to reference sequences of the genes *BRCA1* (NM_007294.3), *BRCA2* (NM_000059.3) and *PALB2* (NM_024675.3) using the Burrows‐Wheeler Alignment tool. Local realignment and variant recalibration were conducted using the GATK (Genome Analysis Toolkit) Best Practices Pipeline. Variants were annotated by annovar software tools (http://annovar.openbioinformatics.org/).

PCR amplification and Sanger sequencing of variants were applied to human gDNA of the available individuals for variant verification and co‐segregation analysis by designing primer pairs (BRCA2‐7007L and BRCA2‐7007R) through the online Primer 3 programme (http://primer3.ut.ee/) in the corresponding *BRCA2* genome (Table 1). A PCR product with 553 bp for *BRCA2* was amplified using gDNA as a template. The PCR products were directly subjected to sequencing with the Sanger method on an ABI‐3500DX sequencer (Applied Biosystems Inc, Foster City, CA, USA) using the specific primer BRCA2‐7007L (Table 1).^16^, ^17^ All unrelated, ethnically matched controls were also sequenced using the aforementioned primer, and then, co‐segregation analysis was performed based on the sequencing results and clinical phenotypes in this pedigree.

**Table 1:** The sequences of *BRCA2* PCR primers

Forward primer	Sequence (5′‐3′)	Reverse primer	Sequence (5′‐3′)	Size (bp)
BRCA2‐7007L	tgtactgtgagttatttggtgca	BRCA2‐7007R	ttaactgattcggagcaatttcc	553

### 2.3 Protein structure and bioinformatic analysis

The conserved domains were identified by the online NCBI system for the BRCA2 protein https://www.ncbi.nlm.nih.gov/Structure/cdd/wrpsb.cgi.^18^, ^19^

## 3 RESULTS

We recruited a Chinese breast cancer family, including 3 female patients and 25 healthy individuals (Figure 1, II:3, III:3, III:11, Table 2), from Hunan province in the central south of China. The patient II:3 was diagnosed with breast cancer at the age of 60 and died 4 years later. The proband (Figure 1, proband, III:11, molecular no.: M411) was 41 years old and diagnosed with breast cancer three years ago (Figure 1B‐D). The proband and her sister with breast cancer (Figure 1, III:3) were diagnosed with advanced invasive ductal breast carcinomas. The clinical information of the two patients, including current age, the year diagnosed, unilateral status, treatment strategy, tumour grade and size, lymph node status, recurrence, IHC results for biomarkers and FISH results, is listed in Table 2. By IHC analysis based on biomarker testing of the cancer tissues, the proband's tumour was positive for Ki67, ER, PR and HER‐2, whereas other markers were negative (Table 2). Both patients were initially diagnosed with stage II disease (Table 2). Treatments were used immediately when diagnosed, and the recurrences were not found over three years for the proband (III:11) and five years for her sister (III:3) (Table 2).

**Table 2:** Clinical information summary of patients with breast invasive ductal carcinomas

Mol No.	age (y)	Fa	Lat	Treatment	Grade	Tumour size	Lym	Rec	IHC	FISH
M411	41	39	R	Sur, Che, Rad	II	3.5x3x2	2(15)	N	Ki67(+,30%), ER(+,50%), PR(+0.60%), HER‐2(+), EGFR(−), CK5/6(−), P63(−), CD34(+), D2‐40(+)	No
M415	51	47	R	Che	II	2.7x2.7	NA	N	NA	NA

Abbreviations: Che, chemotherapy; Fa, found age; Lat, unilateral status; Lym, lymph node; Mol No., molecular number; N, not; NA, not available; No, no copy number amplification; R, right breast; Rad, radiotherapy; Rec, recurrence; Sur, surgical treatment; Tumour size, cm.

**Figure 2:**
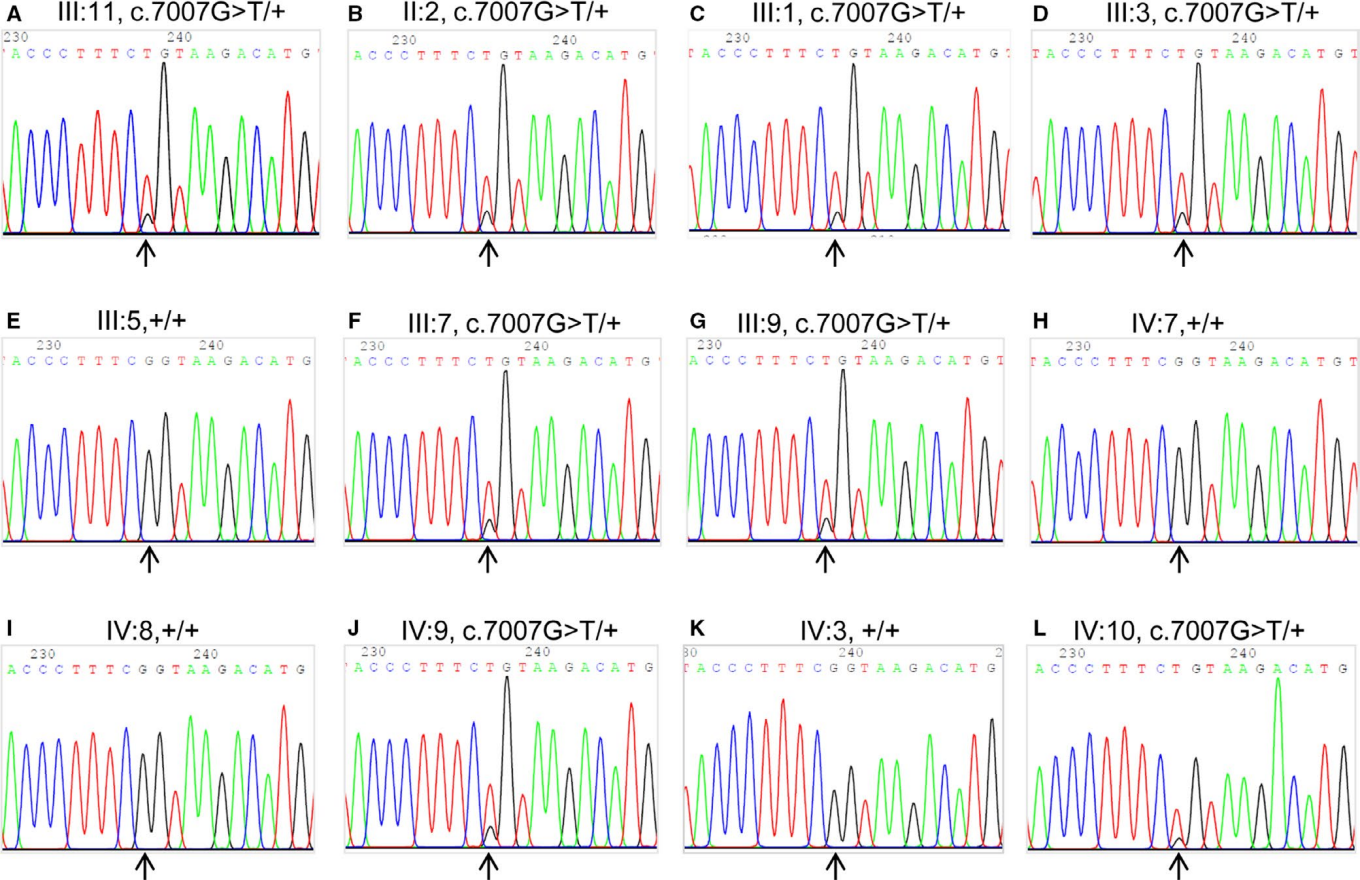
Photogram profiles for *BRCA2* verification by Sanger sequencing. A‐L, indicate the sequencing results for the *BRCA2* gene in the pedigree III:11, II:2, III:1, III:3, III:5, III:7, III: 9, IV:7, IV:8, IV:9, IV:3 and IV:10, respectively. The arrows indicate mutations at the nucleotide positions: c.7007G>T in the *BRCA2* gene. ‘+’: wild‐type allele of *BRCA2*

Blood samples were collected from the family members, and DNA was extracted from it. After sequencing all of the exons and exon‐intron boundaries of the *BRCA1/2* and *PALB2* genes by NGS‐based genetic diagnosis in the proband, we identified a heterozygous missense mutation of the gene *BRCA2*: NM_000059.3: c.7007G>T (p.R2336L) in exon 13, which substituted base T for base G at the base position 7007, leading CGC to be mutated into CTC (c.7007G>T), causing arginine (Arg, R) to be substituted by leucine (Leu, L) (p.R2336L). This variant was then verified by Sanger sequencing in the proband (Figure 2A). PolyPhen‐2 analysis suggested this change was benign (score 0.0011), but MutationTaster revealed the change to be disease causing (score 1), SIFT indicated it was tolerated (score 0.46), and I‐Mutant2.0 for the free energy change value indicated an increase in protein stability (DDG = 0.61 kcal/mol, >0). This variant of *BRCA2*: NM_000059.3: exon 13: c.7007G>T, p.R2336L, is likely pathogenic. By searching the ExAC and HGMD databases, this variant was found to be novel. The pathogenic aspects of this mutation are presented in Table 3.

**Table 3:** Characteristics of BRCA2 variant and analysis of predicted protein structure and disease‐causing effects

Gene	Exon	Variation				Polyphen‐2	Mutation Taster	I‐Mutant2.0	SIFT	ExAC
		Nucleotide*	Amino acid *	Type	Status					
BRCA2	13	c.7007G>T	p.R2336L	Missense	Heter	B (0.011)	DC (1)	DDG: 0.61kcal/mol	T (0.46)	Novel

Note

B, benign; BRCA2, breast and ovarian cancer susceptibility protein 2 (NM_000059.3); c, variation at cDNA level; DC, disease causing; DDG > 0, increase stability; ExAC, the Exome Aggregation Consortium.; Heter, heterozygote; p, variation at protein level; R2336L, leucine substitution conserved arginine at codon 2336; T, tolerated.

* All nucleotide and amino acid are abbreviated according to the International Union of Pure and Applied Chemistry (IUPAC).

Sequencing of DNA materials from nine other female members in this family including the proband's sister with breast cancer was also performed. The representative results by Sanger sequencing are shown in Figure 2A‐J. In addition to proband III:11 and her sister III:3 who were affected with breast cancer, another five women in this family, including the proband's mother, three sisters and one niece, who all appeared to be normal, also carried the same heterozygous, missense mutation of the gene *BRCA2*: c.7007G > T. The other 3 women in this family with normal phenotypes carried two wild‐type alleles (Figure 2). DNA from III:13 was not available. Sequencing of DNA materials from male members of this family was also performed and revealed that IV:3 had wild‐type alleles of *BRCA2*, whereas IV:10 had the same heterozygous mutation of *BRCA2*: c.7007G>T (Figure 2K,L, respectively). One hundred unrelated, healthy individual controls did not show this variant by Sanger sequencing (data not shown).

**Figure 3:**
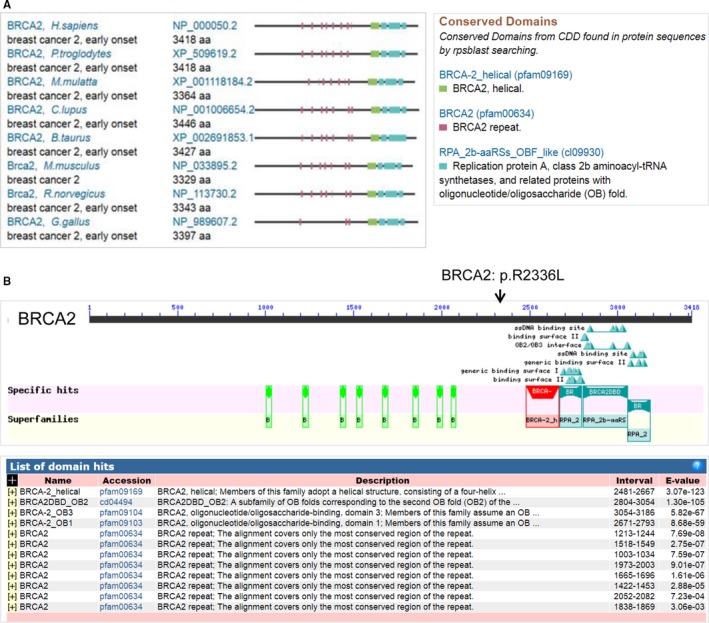
BRCA2 conservation (A), human BRCA2 structure (B, up panel) and conserved domains (B, bottom panel). The amino acid mutation position BRCA2: p.R2336L is indicated by an arrow

Searches of the Conserved Domain Database (CDD) in NCBI were executed. Comparing the human BRCA2 protein to nine other species indicated that it is highly conserved in the chimpanzee, rhesus monkey, dog, cow, mouse, rat and chicken (Figure 3A). Comprehensively, this study shows that the heterozygous variant of *BRCA2*: c.7007G>T (p.R2336L) might cause breast cancer in this Chinese pedigree (Figure 3B, arrow).

## 4 DISCUSSION

The *BRCA2* gene, also known as *BRCC2*, *BROVCA2*, *FAD*, *FACD*, *FAD1*, *GLM3*, *FANCD*, *PNCA2*, *FANCD1* and *XRCC11*, functions as a tumour suppressor gene and is involved in repairing damaged DNA, and mutations of it are associated with diseases including fanconi anaemia, complementation group D1, fallopian tube cancer, primary peritoneal cancer, ovarian cancer and breast cancer.^20^ Numerous reports have indicated that inherited germline mutations in the *BRCA1* 9, 21, 22 and *BRCA2* genes^10^, ^23^, ^24^, ^25^ result in an increased risk of developing breast or ovarian cancer sometime during their life‐times. The prevalence and clinical outcome of germline mutations in the *BRCA1/2* and/or *PALB2* genes in breast cancer patients in different ethnic groups, including populations from Turkey,^26^ Lebanon,^27^ Japan,^28^ Mexico,^29^ China,^30^, ^31^, ^32^ etc, have been widely reported recently. By analysing large numbers of Chinese hereditary breast and ovarian cancer patients and Caucasian patients, Bhaskaran et al^33^ recently revealed that germline variations in the *BRCA1/2* genes are highly ethnicity‐specific. Thus, identification of novel pathogenic germline *BRCA2* variants in familial Chinese breast cancers is necessary.

In the current study, NGS‐based genetic diagnosis 14, 34, 35, 36 was used to identify a novel heterozygous missense mutation of the gene *BRCA2*: c.7007G>T, p.R2336L, in a Chinese breast cancer family. This gene is likely pathogenic, enriching its known mutation spectrum. Two breast cancer patients including the proband, her elder sister III:3 and another five women (the proband's mother, three sisters and one niece) in this family whom appeared to be normal through August 2019 also carried this heterozygous, missense mutation of *BRCA2*: c.7007G>T. Because of financial and medical constraints, the carriers declined to go to hospitals for breast cancer screening. Nevertheless, it may be used as a biomarker for the *BRCA2* variant (c.7007G>T) in this family.

Women carrying *BRCA1/2* mutations have an increased life‐time risk of developing breast cancer, and multiple risk factors, such as sex, age, ethnicity, environmental factors, diet and lifestyle, affect the penetrance, progression and development of breast cancer.^7^, ^37^, ^38^, ^39^ Antoniou et al,^40^ for example, reported that the breast cancer risk for female carriers of *PALB2* mutations was increased by eight to nine times among those younger than 40 years of age, six to eight times among those 40 to 60 years of age and five times among those older than 60 years of age, when compared to the general populations. Environmental factor, being overweight/dietary choices, having a first child at an older age, few or no childbirths and the lack of or only a short period of breastfeeding should increase the risk or penetrance for developing breast cancer in this family.^7^, ^41^, ^42^ Therefore, it is not surprising that the proband's mother, as a carrier, is still unaffected at 76 years of age, as she gave birth to seven children with her first baby at the age of 22, and they were all breastfed. But the other carriers in this family should be warned that they have a higher risk of breast cancer, especially the proband's 9‐year‐old niece. In this regard, this knowledge may provide viable strategies for breast cancer prevention through avoiding some of the other risk factors such as those aforementioned. Thus, genetic counselling and long‐term follow‐up should be provided for this family, especially the patients and carriers of this variant of *BRCA2*: c.7007G > T (p.R2336L).

Offspring of individuals with a germline *BRCA1/2* or *PALB2* pathogenic variant have a 50% chance of inheriting the variant. The male individuals IV:1, IV:2, IV:3, IV:4 and IV:10 have a 50% chance of inheriting the c.7007G>T variant, and if they have a daughter, she may be at high risk of breast cancer. In addition, men who carry *BRCA2* variants also have an increased risk of developing breast cancer.^20^ Thus, we collected samples from men, and Sanger sequencing revealed that IV:3 had wild‐type alleles of *BRCA2*, whereas IV:10 carried the heterozygous missense mutation of the gene *BRCA2*: c.7007G>T (Figure 2L). Unfortunately, the male individuals IV:1, IV:2 and IV:4 could not be tested. Prenatal testing is possible for pregnancies if a pathogenic variant is known; however, requests for prenatal diagnoses of mutations linked to breast cancer are not common and require careful genetic counselling.

In conclusion, we have successfully identified a novel germline heterozygous, likely pathogenic variant of *BRCA2*: c.7007G>T (p.R2336L) in a Chinese family with breast cancer by NGS‐based genetic diagnosis, enriching its known mutation spectrum. Genetic counselling and long‐term follow‐up should be provided for this family and in other patients and carriers carrying this *BRCA2* variant: c.7007G>T (p.R2336L).

## AUTHORS’ CONTRIBUTIONS

JC, JiF, PT, XD, CW, JP and HC recruited patients, collected samples and conducted experiments. JF and MAK wrote the manuscript; and JF revised the manuscript. JF supervised the project. All authors reviewed and approved the manuscript.

## ETHICAL APPROVAL AND CONSENT TO PARTICIPATE

The study was approved by Southwestern Medical University, and written informed consent was obtained from all patients, in compliance with the recommendations of the Helsinki Declaration.

## CONSENT FOR PUBLICATION

Written informed consent was obtained from the participants for publication of their medical data and images.

## CONFLICTS OF INTEREST

The authors declare that they have no conflicts of interest.

## Data Availability

All data used to support the findings of this study are available from the corresponding authors upon request.
